# The hearing screening experiences and practices of primary health care nurses: Indications for referral based on high-risk factors and community views about hearing loss

**DOI:** 10.4102/phcfm.v10i1.1848

**Published:** 2018-10-10

**Authors:** Nasim B. Khan, Lavanithum Joseph, Miriam Adhikari

**Affiliations:** 1 Discipline of Audiology, University of KwaZulu-Natal, South Africa; 2 School of Clinical Medicine, University of KwaZulu-Natal, South Africa

## Abstract

**Background:**

In South Africa, primary health care is the first point of contact with the health system for at least 85% of the population, yet early hearing detection and intervention continues to be elusive in these settings. Nurses at community level may, therefore, be missing an opportunity to identify prelingual infants with hearing losses and alter their developmental trajectory.

**Aim:**

To determine primary health care nurses’ experiences, practices and beliefs regarding hearing loss in infants.

**Setting:**

The study was conducted in the eThekwini District of KwaZulu-Natal, South Africa.

**Methods:**

A descriptive survey was used with quantitative methods of analysis. Fourteen primary health care clinics from the eThekwini district were selected, from which 75 nurses participated by completing a self-administered questionnaire.

**Results:**

At least one-third of primary health care nurses had never screened a child for hearing loss, and most clinics did not have access to basic hearing screening equipment or materials. Only 49% of nurses had access to an otoscope, while 31% used the Road to Health Development screener to check for hearing loss. None of the clinics had access to an otoacoustic emission screener nor the Swart questionnaire. Although nurses reported that they would refer to audiology services for some of the risk factors, as indicated on the Joint Committee on Infant Hearing (JCIH) 2007 list, they were less likely to refer if the child was in a neonatal intensive care unit (ICU) longer than five days, had neurodegenerative disorders, meningitis, hyperbilirubinaemia requiring blood transfusion or were undergoing chemotherapy. Less than a third of nurses always referred if the child displayed additional non-JCIH risk factors or those pertinent to the South African context. Approximately 38% reported that communities believed that hearing loss could be because of some form of spiritual or supernatural causes.

**Conclusion:**

This study demonstrates that hearing screening and referral practices at primary health care clinics need to be strengthened. Nurses need to be capacitated to conduct basic screening, make necessary referrals, provide information to caregivers and understand community beliefs about hearing loss in order to counsel caregivers appropriately and facilitate the process of early hearing detection and intervention.

## Introduction

Hearing loss is a commonly occurring birth defect in developing countries.^1^ Unidentified hearing impairment especially in infancy has been associated with adverse and permanent deficits in speech and language development, academic achievement and social, emotional and cognitive development in children.^2,3^ The Joint Committee on Infant Hearing (JCIH) position statement, year 2007, on early hearing detection and intervention (EHDI)^4^ states that the hearing of infants must be screened by 1 month of age. If problems are identified and infants have a refer result, a diagnostic evaluation needs to be completed by no later than 3 months of age, with interventions by no later than 6 months that include various forms of habilitation, rehabilitation or educational programmes.^4^ The position statement of the Health Professions Council of South Africa (HPCSA) on EHDI^5^ supports the above, with slight contextual adjustments in relation to the timeframes for screening, diagnosis and intervention.^6^ In addition to the initial screening, the infants demonstrating risk indicators for delayed onset and progressive hearing loss must receive ongoing surveillance by caregivers who are informed about the risks and the communication development milestones that need to be observed.^5,7^

In South Africa (SA), primary health care (PHC), which is mainly nurse-driven, is the first point of contact with the health system for at least 85% of the South African population.^1^ The World Health Organization (WHO) states that PHC is accepted as the best model for delivering basic health care, being an appropriate and a cost-effective means of improving the population’s health and a driving force in promoting health care.^8^ However, limited attention has been afforded to hearing health, especially in primary care settings in developing countries.^9^ The inadequate provision of good primary ear and hearing care throughout most of sub-Saharan Africa, not only exposes patients to potentially life-threatening complications from ear disease, but results in the common problems of chronic ear disease and disabling deafness.^9^

There are too few audiologists available in SA to provide an accessible and equitable service at community level. A total of 1800 speech and hearing therapists, audiologists and/or speech therapists, mainly urban-based within the private sector, service a population of over 53 million people in the country, making access to services difficult for most, particularly in rural areas.^5,10,11^ The effectiveness and success of programmes that provide for the early detection and intervention of children with hearing loss is contingent upon an interdisciplinary team approach.^5^ Team members involved should include audiologists; speech-language therapists; nurses; paediatricians; ear, nose and throat specialists (ENTs), community workers and families.^5^ This multidisciplinary or interprofessional method of shared knowledge and skill for a common goal, such as EHDI, will advance hearing health outcomes, while pooling the necessary resources.^12^ Findings of research conducted in SA indicate that hearing screening is not happening at the PHC clinics, where most caregivers take their infants for health care services.^13^

There is limited knowledge about the extent to which the assessment of developmental milestones is part of the medical protocol in developing countries.^14^ In Cambodia, as an example, it was found that nurses are mainly trained to care for patients presenting with acute illness and to monitor the growth of children, with many lacking training in child development.^14^ In SA, medical attention is usually sought because of acute illness rather than developmental disabilities and delays, which, together with practitioners’ frequent lack of awareness, results in the at risky behaviours being overlooked.^15^ Similarly, in a study conducted in Brazil, it was found that most nurses are not trained in the importance of preventing hearing disorders, the identification of risk factors and referring for diagnostic testing.^16^ In another study conducted in Brazil, over 86% of nurses had not received training and information about referrals for hearing testing in infants.^17^ However, in a study conducted in Nigeria, hearing screening by PHC nurses was found to be successful, as most children presented for their immunisation visits irrespective of where they are born.^18^ This is advantageous, as it allows the identification of infants who were not screened at birth because of the limited opportunities for mothers to access universal newborn hearing screening (UNHS), especially in developing contexts. This also allows for the identification of progressive or late onset hearing losses and provides opportunities for caregivers to be sensitised about hearing loss. It can be a measure that is repeated each time the child is immunised, thus also ensuring that any child who has a hearing loss may be identified and loss to follow-up minimised.^19^ Identifying hearing loss by aligning mothers and/or primary caregiver reports (caregiver-driven) and nurse identification (systems-driven) during immunisation visits may be a viable option in SA, which has a reasonably good immunisation coverage rate, although its timelines need to be improved.^20^

A study conducted in the United States of America showed that nurses trained in ear care can substantially reduce treatment costs, antibiotic usage and referrals to ENT specialists and general practitioners.^21^ In the absence of hearing screening programmes at the PHC level of care, infants with hearing loss may go undetected for many years, leading to adverse consequences. This age group is particularly vulnerable, as they are unable to communicate using speech and language and cannot answer questions related to their hearing status. The only ‘formalised’ screening for hearing loss by the South African Department of Health (DoH)^22^ that is generally available, is the voice test and the Swart questionnaire.^23^ It is important that PHC nurses be very familiar with both these tests to ensure early identification and intervention of hearing loss. According to the DoH,^22^ it is protocol for nurses to complete a subjective screening test, such as the Swart questionnaire^24^ or the voice test, with children suspected of having a hearing loss. However, in the absence of knowing their hearing status, such infants may be overlooked.

Despite the shortage of nurses in the health system and their extensive workloads,^25^ they are in an ideal position to screen for hearing loss. In addition, nurses understand the cultural and linguistic backgrounds of the communities they work in and are knowledgeable about some of the high-risk factors associated with hearing loss.^26^ Nurses can also serve as intermediaries between caregivers, the doctor and the referral source.^26^ Furthermore, parents’ or families’ myths, cultural beliefs and societal practices related to the causes and treatment of hearing loss also influence their choice of practitioners and health-seeking behaviours, irrespective of educational level or social standing, and can further delay identifying and intervening for this disability.^18,27^

Parent and caregiver education is essential in a multicultural setting such as SA, because of the cultural and social stigmatisation that a disability carries.^25^ It is also important to note that societies and cultures have different understandings about what is considered to be normal and disordered,^28^ making it important for information that is given to the parents and caregivers to be culturally sensitive and appropriate. Additionally, in a diverse and multicultural context, such as SA, the manner in which parents are informed about hearing screening, the available treatment options and who gives them the information is very important.^26^ Managing and monitoring hearing is within the scope of practice for PHC nurses, as the South African Nursing Council^29^ notes that they are responsible for maintaining the sensory functions in patients, which includes hearing. Maintaining hearing can be effectively achieved by implementing hearing screening and detection in PHC clinics.

Another area that has been included in the scope of practice for nurses is promoting health and counselling with individuals and groups of people.^29^ This indicates that nurses play an important role in educating mothers and caregivers about pathologies and risk factors related to hearing and hearing loss, as well as counselling patients towards appropriate referrals and keeping appointments that have been made. As nurses execute their role in the overall monitoring of the general health, development and well-being of infants, families become more aware of typical development, including auditory development.^4^ In the absence of formal screening, nurses need to provide mothers and/or caregivers with information and counselling about hearing loss and make timeous referrals on the presentation of high-risk factors. The DoH contends that the nurse’s role should encompass reviewing the child’s medical records, interviewing the caregiver and completing a physical examination that includes otoscopy and checks for otitis media, conducting a throat examination, checking for neck stiffness and examining the mastoid for pain. They should also complete a hearing screening evaluation, record the results and refer appropriately.^22^

In a study conducted by Thandrayen and Saloojee^30^ on the quality of care offered to children attending PHC clinics in Johannesburg, it was found with concern that PHC nurses often failed to appropriately assess a child suspected with meningitis, which is a risk factor for hearing loss. Petrocchi-Bartal and Khoza-Shangase^31^ conducted a study at immunisation clinics in the Northwest and Gauteng provinces with 30 PHC nurses and found that there were no formalised newborn and/or infant hearing screening programmes at any of the clinics. They cited key concerns related to limited training received and budgetary, human resource and equipment constraints. While otoscopes were available, they were only used by 76% of nurses to sometimes conduct otoscopic examinations in children under five years of age, mainly if they presented with upper respiratory tract infections. There was also an inconsistent application of the hearing screening assessment protocol stipulated in the Road to Health Card (RtHC), with the guidelines further stipulating that results obtained for hearing screening be documented on the card to ensure continuity of care for children.

The HPCSA position statement recommends that each district health system in SA use an integrated information system to manage data.^5^ One of the roles of the PHC nurse is to record the hearing screening results so that important patient information can be accessed by both the parents and other staff members. Joubert and Casojee^32^ found that this was not deemed to be necessary information by PHC nurses, who did not adhere to hearing screening or record-keeping practices. The lack of urgency and low priority given to hearing impairment at PHC level may be because of issues related to poverty and the burden of life-threatening diseases, such as the human immunodeficiency virus (HIV) and/or acquired immune deficiency syndrome (AIDS) and tuberculosis.^33,34^

There is a paucity of literature in SA related to how PHC nurses identify and engage with children with hearing loss who attend clinics. Their referrals, based on high-risk factors, as well as information and education provided to caregivers, given the beliefs about the causes of the condition at community level, need clarification. Information obtained will also guide appropriate education, awareness and training programmes for nurses and develop appropriate guidelines for detecting hearing loss in prelingual infants at PHC clinics.

The aim of this study was firstly to determine hearing screening experiences, methods and approaches used by PHC nurses to screen for hearing loss in prelingual infants and secondly to determine their referral practices based on high-risk factors and their views on community beliefs about the causes of hearing loss. The findings will have implications for managing prelingual infants with hearing loss at community level.

## Research method and design

A descriptive survey design was used with quantitative methods of analysis to enable the researchers to make inferences and learn about a large population by surveying a smaller sample.^35^

### Study population

The study participants were PHC nurses working within the eThekwini district in KwaZulu-Natal province, which has a total of 112 PHC provincial and municipal clinics located within it. In SA, the provincial government is responsible for the provision of health care services; however, in some large municipalities, there are municipal clinics that also provide PHC services. This is made possible through a bilateral agreement between the provincial department and the municipality,^36^ with the provincial health department paying the municipality to render these services.

### Sampling method

A multistage (two-stage) sampling strategy was used, firstly to select the clinics and secondly the participants. Based on the estimated numbers of nurses in the facilities, 12 provincial and 18 municipal clinics were selected through simple random sampling. The second stage involved randomly recruiting a minimum of four nurses per clinic to achieve the proposed sample size of 120 from 30 clinics. Nurses of all races, ages (above 18 years), genders and levels of qualification were included, while student nurses were excluded because of their limited work experience.

### Sample size

Of the 30 clinics identified, only 14 agreed to participate in the descriptive survey, including the one pilot site. Of the 74 questionnaires that were completed, nine were discarded because of incomplete responses. Another 10 from the pilot were added to the main study, as there were no changes to the questionnaire. The total number of questionnaires accepted for analysis was 75 of the anticipated 120 PHC nurses. These came from eight provincial clinics with 51 nurses, and six municipal clinics with 24 nurses. Some of the reasons cited for non-participation of clinics included staff shortages, that they did not see children with hearing loss, that they declined to participate, and two clinics lost the forms.

### Description of the sample

The majority of participants were female 85% (*n* = 64), with 48% (*n* = 36) being between 36 and 50 years and 28% being over 50 years of age. A diploma in general nursing level 6 was held by almost half the nurses (43%, *n* = 28), with most (77%, *n *=* *58) respondents being professional nurses, having received their education at various nursing colleges within the province. Approximately 50% (*n* = 38) of the nurses had less than 10 years of experience working in PHC, with 36% (*n* = 27) having over 15 years’ experience.

### Data collection instrument

The data collection instrument was developed by the researcher based on the literature and was adapted from the studies by Moodley and Storbeck^26^ and Joubert and Casoojee.^32^ The risk factors were obtained from the JCIH list of high-risk factors (JCIH 2007),^5^ HPCSA EHDI position statement (HPCSA 2007) and Olusanya.^2^ The questionnaire consisted of three sections: Section A obtained demographic information (eight questions), and Section B (Objective 1) explored how the nurses screen children with hearing loss and their experiences (nine questions). It is important to understand current experiences and methods used by nurses to identify hearing loss at PHC and the challenges faced in order to improve best practice.^37,38^ This includes availability of screening methods, recording and referral practices and information provided to caregivers. Section C included referrals based on high-risk factors (one question with 25 subquestions) and one open-ended question about the views of nurses about community beliefs regarding the causes of hearing loss. In the absence of UNHS, it is essential for nurses to be aware of the high-risk factors for hearing loss to facilitate referrals for further testing^14^ and conduct routine checks, especially for late onset or progressive hearing loss.^18^ Furthermore, nurses need to understand the community beliefs about the causes of hearing loss to enable them to appropriately inform and educate caregivers about the risks and interventions. Studies by a number of authors^39,40^ indicate that mothers or caregivers may hold superstitious beliefs about the causes of hearing loss that can delay intervention, with education having been found to be effective in modifying health-seeking behaviour and reducing non-compliance in developing contexts, despite low education and literacy levels.

### Procedure

All ethical considerations relevant to the study were adhered to, including signed informed consent, voluntary participation and anonymity. Participants were given two weeks to complete the questionnaires and an additional week in order to improve the response rate. A pilot study was conducted with 10 nurses from one clinic in the eThekwini district to ensure reliability and validity. Cronbach’s alpha was used to determine the internal consistency and reliability of the items contained in the survey questionnaire. A score of 0.887 (*n* = 56 items) was obtained, indicating good internal consistency. The data was processed and analysed using SPSS version 23, while NVivo 11 was used for the open-ended question about nurses’ views of community beliefs regarding the causes of hearing loss, allowing for qualitative analysis.

## Results

The results are presented with respect to the two study objectives of, firstly, hearing screening experiences, methods and approaches used by PHC nurses and, secondly, PHC nurses’ reported referral practices based on high-risk factors for hearing loss and views of the community pertaining to hearing loss.

### Hearing screening experiences, methods and approaches used by primary health care nurses

#### Hearing screening experience

Of the 75 participants, 33% (*n* = 25) had never screened a child nor identified a hearing loss. Of the 67% who had screened children, only 28% (*n* = 21) had screened over 20 children with a possible hearing loss, while the remainder had screened less than 20 in their years of working in a PHC setting. The chi-squared test showed a significant association (*p <* 0.001) between the years of experience as a PHC nurse and the number of children screened. Recently qualified nurses with fewer than 10 years of work experience tended to screen more children for hearing loss. Half the participants (55%, *n* = 41) felt that the best time to screen was every time a child attends the clinic, followed by when they come in for their immunisation checks (23%, *n* = 17). No significant association (*p* = 0.38) was noted between those who had or those who had never screened, and the best time to screen. Most nurses (84%, *n* = 63) used the RtHC to record patient information, while the others used the general patient files. In general, when a hearing loss or speech and/or language problem was suspected, referrals were made to various health professionals, including audiologists, ENT specialists, the General Practitioner at the district hospital or clinic and paediatricians.

#### Health information and education provided to mothers and/or caregivers

**FIGURE 1:**
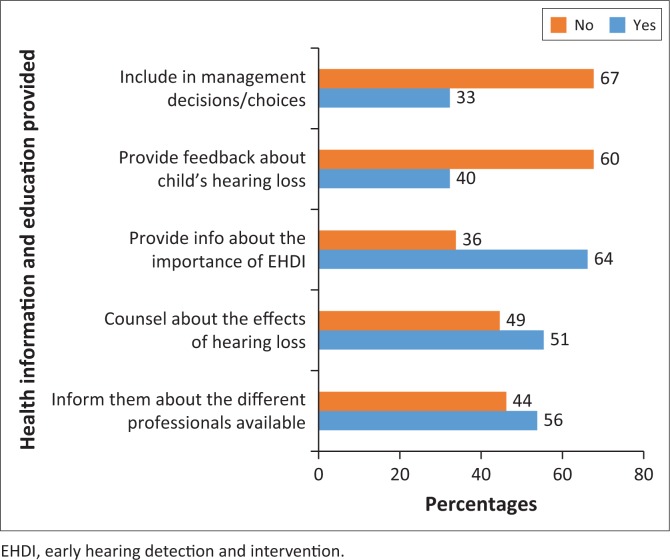
Health information and education information provided to caregivers.

Most nurses 64% (*n* = 48) indicated that they provide information about the importance of EHDI to parents or caregivers, while 51% provided information about other professionals and counselling on the effects of hearing loss (Figure 1). Forty per cent (*n* = 30) of the nurses provided feedback to the caregivers about the child’s hearing loss and a third included them in making decisions about their management options.

#### Screening equipment and strategies

**FIGURE 2:**
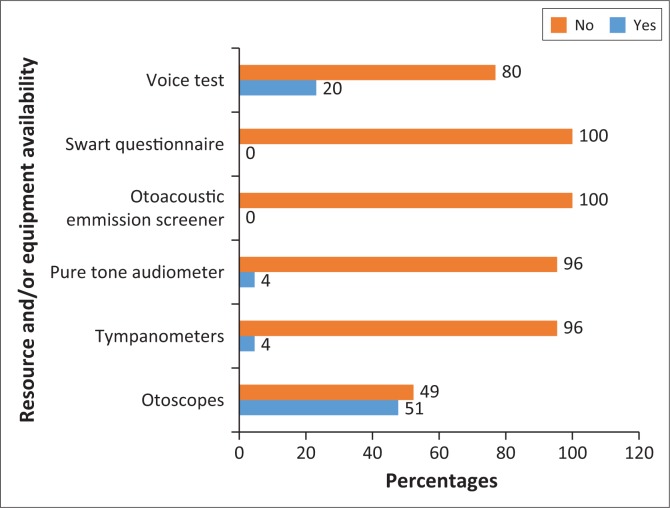
Resource and/or equipment availability.

Regarding the availability of screening equipment, only 49% (*n* = 37) indicated that otoscopes were available in 14 clinics, with two to three being available in each clinic, one clinic reporting only having one otoscope and two clinics having none. Three participants had tympanometers and pure tone audiometers, and 20% (*n* = 15) had the voice test available, with none having the Swart questionnaire (Figure 2).

Regarding their use of any developmental screening charts to assess for hearing, of the 63 who answered this question, 63% (*n* = 47) indicated that they did use them, while only 25 participants indicated exactly what they used to screen: 92% (*n* = 23) used the RtHC that has a developmental screener, one participant spoke to the child and one used a general screener.

### Primary health care nurses self-reported referral practices based on high-risk factors for hearing loss and views of the community pertaining to the causes of hearing loss

#### Self-reported referral practices based on high-risk factors for hearing loss

Risk factors were categorised into four groups: (1) those using the JCIH (2007)^5^ list of high-risk factors, (2) risks emerging from regions in sub-Saharan Africa (Olusanya^2^), (3) known non-JCIH risk factors (Olusanya^2^) and (4) SA context risk factors^4^. The responses are presented in Tables 1 and 2 using percentages, means and standard deviations. Participants were given a four-point rating scale to respond to: (1) always refer, (2) refer most times, (3) sometimes refer and (4) never refer. The mean rating and standard deviation for each of the responses for each risk factor was calculated.

**TABLE 1:** Participants’ self-reported referrals based on the Joint Committee on Infant Hearing 2007 high-risk factors (*n* = 75).

Risk factor	Always refer (1)	Refer most times (2)	Sometimes refer (3)	Never refer (4)	Mean	Standard deviation
Caregiver concern	69.3	22.7	5.3	2.7	1.41	0.718
Syndromes	69.3	4.0	13.3	13.3	1.71	1.136
Family history	68.0	17.3	9.3	5.3	1.52	0.875
Craniofacial anomalies	65.3	10.7	10.7	13.3	1.72	1.110
Head trauma	58.7	18.7	10.7	12.0	1.76	1.063
Neurodegenerative disorders	49.3	12.0	24.0	14.7	2.04	1.156
Maternal infections	46.7	24.0	12.0	17.3	2.00	1.139
Meningitis	41.3	24.0	21.3	13.3	2.07	1.082
Hyperbilirubinaemia	33.3	21.3	13.3	32.0	2.44	1.255
Chemotherapy	34.7	17.3	16.0	32.0	2.45	1.266
NICU > 5 days	29.3	18.7	24.0	28.0	2.51	1.190

NICU, Neonatal intensive care unit.

**TABLE 2:** Participants’ self-reported referrals based on high-risk factors not listed by the Joint Committee on Infant Hearing 2007 (*n* = 75).

Risk Variables	Always refer (1)	Refer most times (2)	Sometimes refer (3)	Never refer (4)	Mean	Standard deviation
**Emerging risks**						
Maternal hypertension	27.7	16.9	30.8	24.6	2.45	1.166
Under-nutrition	27.7	13.8	26.2	32.3	2.59	1.231
Unskilled birth attendant	26.2	10.8	26.2	36.9	2.71	1.217
Non-elective C-section	20.0	9.20	23.1	47.7	2.92	1.194
**Known non-JCIH risks**						
Sickle cell anaemia	26.2	15.4	21.5	36.9	2.68	1.243
Consanguinity	18.5	6.20	20.0	50.4	3.08	1.194
**SA context risk factors**						
Recurrent otitis media	62.9	6.20	15.4	9.2	1.63	1.024
Low Apgar score	46.2	24.6	15.4	13.8	1.93	1.070
Very low birthweight	38.5	15.4	32.3	13.8	2.20	1.127
Maternal substance abuse and/or alcohol	36.9	21.5	24.6	16.9	2.19	1.135
Birth asphyxia and/or hypoxia	32.3	13.8	36.9	16.9	2.33	1.107
Child HIV exposed	26.2	24.6	12.3	36.9	2.60	1.230

HIV, human immunodeficiency virus; JCIH, Joint Committee on Infant Hearing; SA, South Africa.

Over half of the nurses reported that they would always refer when they encountered at least five of the JCIH 2007 list of high-risk factors for hearing loss (Table 1). These included caregiver concern about speech and language development, family history of hearing loss, syndromes associated with hearing loss, craniofacial anomalies and head trauma.

There were fewer referrals made for neonatal ICU admission of more than five days, hyperbilirubinaemia requiring blood transfusion, neurodegenerative disorders, meningitis and chemotherapy. Less than a third of nurses always referred if the child displayed risk factors relevant to developing countries or known non-JCIH risk factors (Table 2). Most nurses would always refer if otitis media was evident with fever, making fewer referrals for a low Apgar score, low birthweight, birth asphyxia, maternal substance abuse and child exposed to HIV and/or AIDS (Table 2).

#### Views of the community pertaining to the causes of hearing loss

**FIGURE 3:**
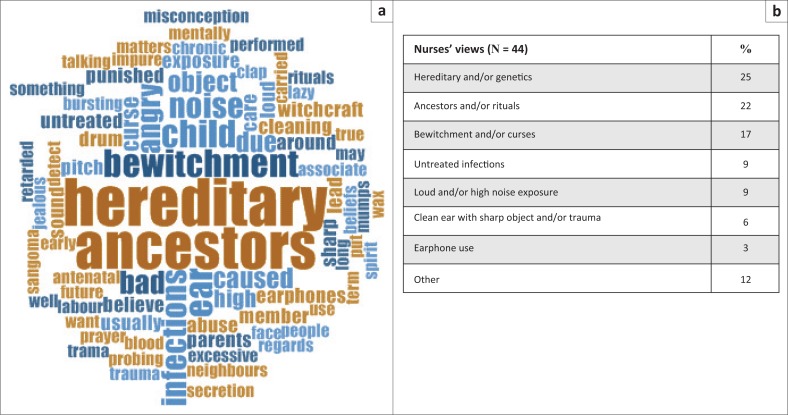
(a) Primary health care nurses’ views on community beliefs about the causes of hearing loss represented in a word cloud. (b) Word frequency counts.

Nurses were given an open-ended question to obtain their views about the beliefs of the community pertaining to the causes of hearing loss. Of the 75 nurses, 44 answered this question, with the results being thematically analysed and depicted as a word cloud, with word frequencies (Figure 3) showing some emerging themes. The nurses felt that the communities’ beliefs regarding the causes of hearing loss included genetics or hereditary (25%) (*n* = 11); loud noise or high noise exposure or high-pitched noise (9%) (*n* = 4), untreated infections (9%) (*n* = 4), trauma or parents hitting child on the ear or cleaning one’s ear with sharp objects and/or eardrum bursting (6%) (*n* = 3), earphone use (3%) (*n* = 1 and other causes including mumps, excessive wax, mental retardation and poor antenatal care during pregnancy. Additionally, 39% (*n* = 17) reported some form of spiritual or supernatural causes of hearing loss, which included rituals not being followed, ancestral spirits, angry ancestors, prayers not performed, parents or family members being punished by ancestors, family did not do well with regard to ancestor matters, beliefs that ancestors want something a child may have (stated by one participant without elaboration), ancestral spirit wants the child to be a sangoma in the future, curse by ancestors and punishment by ancestors because of rituals not being carried out. They also mentioned bewitchment, curses, witchcraft, jealous neighbours, the child being bewitched and blood being impure (17%, *n* = 7).

## Discussion

The PHC nurses had very few encounters with children with hearing loss, and the methods used to screen hearing for the condition at community level were inadequate. Similarly, in a study conducted in Nigeria,^41^ 38.7% of nurses only indicated one previous encounter with an infant with hearing loss. A study in the Gauteng and Northwest provinces of SA also found that 100% (*n* = 30) of nurses based at immunisation clinics reported that no formal hearing screening was conducted and that the HPCSA screening guidelines were not followed at the PHC level.^13^ The authors contend that it is cause of concern, given that this level of care is most accessible to the majority of people, including mothers or caregivers of infants with possible hearing loss.^13^ The results of the current study indicated that recently qualified nurses screened the most, which could be because of the increased awareness of EHDI programmes in SA.

Over half (54%) of the nurses noted that the best time to screen or identify a possible hearing loss was every time a child attends the clinic or presents for immunisation (22%). However, this is seldom done in practice. Research shows that there are very few opportunities for infant screening in PHC clinics in SA,^42^ which is very alarming. More than 50% of nurses reported that they provided the parents and/or caregivers with information about other professionals who could assist the children with hearing loss, counselling about the effects of hearing loss and information about EHDI services. Fewer nurses explained the child’s hearing loss and provided management options. Caregiver education was found to be successful in modifying health-seeking behaviour and reducing non-compliance in developing contexts, despite low education and literacy levels.^40^

Another South African-based study found that children who had been screened and referred did not always attend the follow-up appointments.^38^ It was deduced that poor attendance was because of parents and/or caregivers not having sufficient knowledge on the importance of hearing in a child’s development. If a refer result is found, nurses should be able to provide the parents or caregivers with immediate counselling on the importance of follow-up appointments and information on possible hearing pathologies.^26^ Parental support, education and participation in EHDI is crucial to ensure follow-up and after-care services for children with hearing loss, as non-compliance with treatment is common, especially in developing contexts.^40^

However, the authors do acknowledge that other factors also play a role, such as accessibility and affordability of services, parental cultural beliefs towards childhood deafness and other priorities that mothers and/or caregivers have to deal with. SA is characterised as a multicultural, multilingual country, making it important for professionals to provide services that are culturally and linguistically appropriate.^28^ Recommendations that are associated with social stigma or offend cultural norms could result in loss to follow-up.^26^ As an example, there is considerable stigma attached to taking relatively healthy young children for hearing testing.^18^ It is essential to enable parents to make informed decisions about their child’s management, making the nurse well positioned to explain the importance of screening, follow-up and intervention.^26^ In whatever format this information is provided, parents should receive information in a responsive and sensitive manner for them to be amenable to accessing follow-up services.^26^ They conclude that nurses are often best able to communicate in a culturally appropriate language to make parents feel relaxed and respected for their choices. However, a study conducted by Arnold et al.^43^ stated that parents felt nurses needed more training regarding what to say to them if their child did not pass the hearing screening.

Based on the non-availability of equipment, it is clear that none of the clinics provided any formal screening. Similar findings were reported by Petrocchi-Bartal and Khoza-Shangase^13^ in clinics in Gauteng and North-West Provinces, where all participants reported not having access to equipment, citing budgetary and human resource constraints. While it was anticipated that clinics would not have access to tympanometers, pure tone audiometers and otoacoustic emission testing, it was a serious cause of concern that 52.3% of the participants indicated that they did not have an otoscope available at some of the clinics, which implies that this examination is not always conducted on children at these facilities. Petrocchi-Bartal and Khoza-Shangase^31^ reported that although all their participants reported having an otoscope, approximately 76% indicated that they only used it on some babies. The PHC package outlines the availability of otoscopes as a basic equipment requirement for the inspection of the external auditory meatus and tympanic membrane, and to prevent and detect hearing loss because of otitis media.^22,31^ The Swart questionnaire^24^ and Voice test are two subjective tests recommended by the DoH.^22^ It is, therefore, cause of concern that 100% of the nurses indicated that they did not have the Swart questionnaire and 76.9% did not have the Voice test. A study on the quality of services provided by clinics in the Johannesburg area also showed that the Swart questionnaire was only conducted 14% of the time and the Voice test only 7%.^44^ This has serious implications for the early detection of hearing loss and its further management.

The lack of EHDI services has been reported by many researchers, and it is often regarded as a low priority and of little importance.^34^ There remains a lack of emphasis on early screening and identifying disability throughout Africa, which results in few referrals and limited interventions.^26^ One of the main challenges is the lack of funding for screening programmes, as emphasis is placed on reducing the high mortality rate from the burden of communicable diseases.^26^ While reducing the high mortality rates from these diseases such as HIV and AIDS and tuberculosis (TB) in SA is important, Moodley and Storbeck^26^ further stated that ‘it is likely that a reduction in the mortality rate could result in an increase in the number of children who survive with disabilities’ (p. 29). This shows the need for funding to be given to screening programmes in order to improve the overall health of the paediatric population in SA.

Every nurse is in a position to make appropriate referrals when hearing problems are detected, as well as to ensure proper record-keeping practices, and should keep a record of the management decisions and referrals made.^22^ Late referrals or no referrals for infants presenting with hearing loss is problematic, as the lack of timeous intervention represents a threat to their future quality of life.^14^ The DoH^22^ guidelines state that PHC nurses are to record the results of the hearing screening completed during an immunisation session on the RtHC.^31^ However, only 54% of nurses in the current study stated that they recorded the results as required, despite this card needing to be shown to the nurse at every PHC clinic visit.^24^ Recording the child’s progress is important, as it creates a partnership between the parent and/or caregiver and the health professional and thus promotes patient care and follow-up appointments.^24^ It is also important to note that constant recording in the RtHC will aid ‘a national information system that meets the requirements for hearing screening record-keeping, as recommended in the HPCSA 2007 position statement’ (p. 27).^32^

The lack of importance given to early screening and identification of disability in Africa may suggest that training and experience in the area of hearing loss is limited.^26^ A study by Kanji and Opperman^6^ found that a large number of participants were not effectively followed up on for hearing screening, and while this may be because of poor return rates it could also be attributed to poor record-keeping practices. Diagnostic follow-up and intervention serve as critical ethical markers in the hearing screening process and provide a means of recording outcomes, such as the overall efficiency and effectiveness of screening programmes.^4,5,45^

Nurses and audiologists, as well as families and community workers, are part of the collaborative interdisciplinary team required to service the population with hearing loss and those at risk to ensure that affected children receive an appropriate quality of service.^5,26^ Many health care professionals are unaware of the significance of appropriate interdisciplinary referrals.^42^ This was also found to be the case in the study by Casojee,^46^ who noted a general lack of compliance with referral protocols. The author thus concludes that this not only presents a stumbling block for health systems development but is also contradictory to the principles of EHDI.^46^ According to the DoH,^22^ nursing staff should keep a record of all patients seen and all referrals made. PHC nurses do not adhere to record-keeping or the referral practices^31^ required by the DoH,^22^ which hinders quality patient care.

Nurses must be aware of the risk factors for hearing loss in order to facilitate EHDI at community level. The HPCSA (2007) states that even if a child passes the screening, if they exhibit risk factors for a progressive, late-onset hearing loss or other auditory pathologies that can lead to a speech and language delay, the child should be monitored, and the parents informed of the risks and provided with information on the developmental milestones. In this current study, nurses indicated that they were less likely to refer if a child was in neonatal ICU for more than 5 days and had low birthweight that was usually related to prematurity or neonatal jaundice. However, a retrospective record review of paediatric cochlea implant recipients conducted by Le Roux et al.^47^ found the most frequent risk factor to be neonatal ICU admission (28.1%) and prematurity (15.1%), with the most common postnatal risk factor being neonatal jaundice. Nurses in this current study were also less likely to always refer if the child had meningitis, yet this was the second most common risk factor for postnatal hearing loss.^47^

Swanepoel, Johl and Pienaar^48^ focussed on the nature of hearing loss and associated risk profile and found that hyperbilirubinaemia was one of the most prevalent risk factors. Other risk factors included birthweight less than 1500 grams, syndromes present, congenital infection, craniofacial defect and bacterial meningitis.^48^ In some countries in sub-Saharan Africa and Southeast Asia, non-JCIH risk factors such as consanguinity and sickle cell anaemia are more common, although they are related to specific racial or ethnic groups.^2^

Emerging risk factors from developing countries, such as Nigeria, include maternal hypertensive disorders in pregnancy, non-elective caesarean delivery, unskilled attendant at delivery and under-nutrition.^2^ However, the author contends that the precise means of how these affect hearing, especially in infancy, is yet to be determined. Less than one-third of nurses in this current study always referred if the child displayed risk factors emerging from developing countries and known non-JCIH risk factors, necessitating greater education and awareness of these risk factors. Children who have disabilities, or present with risk factors for disabilities, also require services from a number of health professionals,^49^ and this needs to be facilitated as early as possible. For the South African context, diseases such as maternal HIV and/or AIDS and TB need to be considered, as these infections can lead to hearing loss because of their association with low birthweight, prematurity, bacterial meningitis, viral encephalitis and cytomegalovirus.^50^ HIV and/or AIDS, as well as TB are, therefore, important risk factors for hearing loss, along with hyperbilirubinaemia, congenital rubella, congenital syphilis and a family history of permanent hearing loss.

In developed countries, however, certain risks have been minimised or eliminated because of improved health services but are still very relevant in developing contexts, such as those related to low birthweight and birth asphyxia, making it essential for early identification procedures to be coordinated for efficient outcomes.^26^ It was encouraging to note that nurses would always refer or refer most times for most of the JCIH risk factors and otitis media. It is evident that they need to be provided with information and education about other high-risk factors still pertinent to the SA context, such as low birthweight, prematurity, asphyxia and exposure to HIV and/or AIDS. Thus, nurses will be able to obtain case history information from the parent or caregiver and, as a result of being aware of the risk factors, will monitor the child’s development during visits for immunisation. They will also use the information to plan and organise appropriate hearing prevention and promotion awareness activities and referrals based on high-risk factors for hearing loss. A study by Aires et al.^17^ revealed that nurses are only aware of some risk factors for hearing loss but that to effectively conduct the preceding activities they will require more information and training.

Furthermore, it is reported that 50% of children with a hearing loss have identified risk factors that present at birth or within the early years of life.^5^ Some of these factors include jaundice, respiratory distress syndrome, neonatal ICU admissions and infections, such as cytomegalovirus.^5^ It can therefore be deduced that the nurses who are aware of children who present with these risk factors in the community clinics, have the opportunity to screen them for a hearing loss. Expanding PHC nurses’ knowledge will enable them to provide more effective services for the children in their clinics. A policy document that examined the skills needed in the PHC system stated that in community health settings, nurses should have knowledge of otitis media.^51^ This is essential, as a large proportion of children present with otitis media and conditions such as HIV and/or AIDS, are a contributing factor towards middle-ear pathologies. The current study found that 62% of nurses did refer if otitis media was present. This is important as poor socio-economic conditions mean that recurrent otitis media is a reality for many children in Africa.^52^ In a study conducted by Biagio et al.^53^ regarding paediatric otitis media at a PHC clinic, the prevalence of chronic suppurative otitis media was 6.6%. Evidence suggests that poorly managed or unmanaged otitis media can lead to permanent hearing loss.^53,54^ Nurses do receive training in the structure of the ear, otitis media, hearing problems and the effects of hearing losses on speech and language development, although its extent is unclear.^46,55^ However, in a study conducted in SA, nurses expressed an interest in learning more about managing hearing loss at PHC level.^55^

Differences in health beliefs, perceptions of illness and healing methods between the patient and the provider can be a barrier to accepting services.^56^ This indicates why it is essential for nurses to provide education and information to mothers and/or caregivers about the causes and risk factors for hearing loss, as well as the importance of EHDI and follow-up. In SA, where peoples’ beliefs are buried deep within their cultures, it is important that health care professionals take cognisance of these beliefs so that they may be incorporated into information dissemination.^39^ De Anrade and Ross^57^ conducted a study on beliefs and practices of Black South African traditional healers regarding hearing impairment and noted some of the causes of hearing loss to be related to ancestors, noise, congenital factors, bewitchment and blood impurities.

As 8 out of 10 Black South Africans, seek services from traditional healers,^57^ it is not surprising that communities tend to share similar beliefs about the traditional and non-traditional causes of hearing loss, as was noted in the current study. There is widespread stigma associated with having a child with a hearing loss, who may be considered as a bad omen who will bring misfortune to the family.^40^ Some communities regard unnatural causes of hearing loss to include sorcery, spirits, ancestors and failure to perform certain rites and rituals.^57^ The current study also found that there were references to ancestors, rituals, bewitchment and curses. According to Olusanya^18^ it is not uncommon for permanent childhood hearing loss (PCHL) to be attributed to supernatural causes or superstitious beliefs. In a study conducted by Swanepoel and Almec^39^ at least 57% of mothers held at least one superstitious belief regarding the cause of infant hearing loss. Parents may thus also make use of spiritual healing or traditional medicine, which can be potentially harmful.^18^ These factors possibly account for caregivers not returning for follow-up appointments.^38^ However, in the current study there was also mention of causes of hearing loss such as genetics, exposure to loud sounds, untreated ear infections, trauma because of sharp objects used to clean the ears and use of earphones. Olusanya, Luxon and Wirz^58^ attribute the poor knowledge displayed by mothers and caregivers to deficiencies in health education during the antenatal period, which needs to be addressed.

## Implications and limitations

Research evidence supports that as the UNHS is unlikely to be attained in the near future in the SA context, other feasible options need to be developed, revisited or even redesigned to ensure that early identification happens at the PHC level. The study limitations included the small sample size, which may affect the generalisability of the findings. In addition, the beliefs of nurses regarding the causes of hearing loss may not be the same as the communities they serve, which may have introduced information bias in the study. The responses were based on nurses’ self-reports of their practices and not their observed practices.

## Conclusion

This study demonstrates that hearing screening and referral practices at primary health care clinics need to be strengthened. Despite the challenges relating to screening for hearing loss, PHC nurses should conduct some form of early hearing detection at community level, given that this is most accessible to most people and where mothers and/or caregivers take their infants for health care. Child health in SA is one of the key imperatives of the PHC package, and while preventative care is currently limited to immunisation against diseases and conducting growth monitoring, it should include developmental screening. In this PHC context, nurses can be given basic skills during their training by members of the multidisciplinary team to manage hearing loss at community level and thereby reduce the frequency and chronicity of ear disease and disabling deafness.
